# Effects of speech tracking method using hearing aids for hearing disorder in older people

**DOI:** 10.20407/fmj.2024-001

**Published:** 2024-08-28

**Authors:** Erina Ito, Saiko Sugiura, Koki Kawamura, Yasue Uchida, Hirokazu Suzuki, Mariko Shimono, Kazuyo Mise, Hitoshi Kagaya

**Affiliations:** 1 Department of Rehabilitation, National Center for Geriatrics and Gerontology, Obu, Aichi, Japan; 2 Kariya Hearing Clinic, Kariya, Aichi, Japan; 3 Department of Otorhinolaryngology, National Center for Geriatrics and Gerontology, Obu, Aichi, Japan; 4 Department of Otorhinolaryngology, Aichi Medical University, Nagakute, Aichi, Japan; 5 Department of Otorhinolaryngology, Teikyo University School of Medicine, Mizonokuchi Hospital, Kawasaki, Kanagawa, Japan

**Keywords:** Cognition, Frailty, Hearing loss, Speech therapy, Speech-tracking method

## Abstract

**Objectives:**

Speech tracking is defined as a complex act or task of listening in which the learner tracks the speech heard and repeats it as accurately as possible while paying attention to incoming contextual information. This ability involves auditory input, speech output, repetition, and divided attention. In this small-scale study, we aimed to use a speech-tracking method to train older adults with hearing loss and examine its effects on hearing loss from multiple perspectives, including hearing handicap, frailty, and neuropsychological testing.

**Methods:**

Auditory rehabilitation was provided to older patients who purchased hearing aids at the clinic and wanted to engage in rehabilitation. The Hearing Handicap Inventory for the Elderly, Nursing Home Hearing Handicap Index, speech-tracking rate, Token Test, Communication Activities of Daily Living, Kihon Checklist, Mini-Mental State Examination, Digit Symbol Substitution Test, symbol search, and word recall were used for assessments before and after rehabilitation using the speech-tracking method. Changes in scores of each assessment item and sub-item were examined using Wilcoxon’s signed-rank test, and changes per question were examined using the sign test.

**Results:**

The speech tracking rate (*p*<0.001), Token Test score (*p*<0.001), and Mini-Mental State Examination score (*p*=0.035) improved significantly after the training. Notably, the speech-tracking rate improved for 31 of the 33 participants, with a maximum increase of 19.6 phrases per minute.

**Conclusions:**

The combination of a hearing aid and the speech-tracking method improved auditory comprehension.

## Introduction

Hearing loss is common in older adults and can lead to social isolation, depression, and impairment in activities of daily living (ADL). Hearing loss in middle age has the most scope for intervention (8%) and the greatest impact on dementia.^1^ Some hearing-impaired older adults show poor discrimination ability^2^ and difficulty communicating, but are unaware of their condition.^3^ They may also have difficulty hearing speech despite wearing hearing aids (HAs). People who do not find HAs effective may also have trouble getting used to wearing them. Additionally, individuals with hearing loss may have difficulty communicating with others regarding important aspects of daily life, leading to decreased quality of life.^4^ Such patients may benefit from a combination of HAs and auditory rehabilitation.^5^ Furthermore, using cochlear implants and HAs for auditory rehabilitation has been reported to reverse social isolation, depression, and poor cognitive function in older patients with varying degrees of hearing loss.^6^

Speech tracking is used for cochlear implant users and is a method for training and evaluating listening and language comprehension skills using sentences.^7^ The speaker reads the text; the listener tracks it; if there are errors, rephrasing, gestures, and text presentation are used until correct tracking is achieved. Speech tracking is a complex activity involving auditory input, speech output, repetition, and divided attention, as well as content comprehension and memory. However, in Japan, HA training has mainly been conducted by fitting HAs. To date, no interventions have been conducted in Japan using speech-tracking methods, and no studies have reported intervention effects. Recently, the Mizonokuchi method of auditory rehabilitation^8^ was developed. This method aims to improve listening ability using a speech-tracking method. According to Shimizu et al.,^9^ approximately 60% of older patients who visited HA clinics presented with pre-frailty or frailty or were in need of nursing care. Therefore, it is important to evaluate frailty in older patients with hearing loss from the perspective of frailty prevention. The purpose of this study was to investigate the usefulness of auditory rehabilitation for hearing-impaired older adults by comparing the changes in hearing and the presence of frailty and neuropsychological examination results before and after auditory rehabilitation.

## Methods

### Patients

This study included 43 older patients who attended our ear-nose-throat (ENT) outpatient HA clinic between April 2020 and October 2021 and subsequently underwent auditory rehabilitation to maintain or improve their hearing. Among them, eight patients could not complete the rehabilitation because of illness or a request to discontinue, and two patients were excluded because of missing data. Therefore, 33 patients were included in the analysis.

Patients were included if they were native Japanese speakers aged 65 years or over, with maximum speech intelligibility ≤70% and some difficulty using an HA. Specifically, they lived alone and had limited conversational skills, were unable to use HA on a daily basis, and had difficulty managing their HA because of a dead battery. There were no exclusion criteria.

Twenty patients had a history of HA use, whereas 13 patients had no history of HA use. The subjects ranged in age from 66 to 91 years, had an average hearing level of 52±11 dB (measured at 0.5, 1, 2, and 4 kHz) and an average speech discrimination score of 71±18% when using headphones. Among the patients, 20 were men and 13 were women. None of the participants had been diagnosed with cerebrovascular disease or dementia, and none had communication difficulties caused by problems other than hearing loss. None of the patients used nursing care insurance or nursing care services. Seven of the patients lived alone, and 26 had family members living with them. Auditory rehabilitation methods, including the training frequency and exercises, were explained to the patients by ENT doctors and speech language-hearing therapists (SLHTs).

### Intervention

Auditory rehabilitation was performed using speech tracking in accordance with the Mizonokuchi method.^10^ We used 12 texts on health literacy for older adults, including topics such as good habits for the brain, hearing impairment, and dysphagia. A different text was used each time, and the sentences, separated into phrases, were read by the speaker then repeated by the listener to assess how many times the patient could recite the speech in 5 min. Patients who did not finish reading the text in 5 min were asked to continue speech tracking until the end. At the beginning and end of the training, the same text was used to assess changes in the 1-min speech-tracking rate. The following difficulty levels were used: 1) not showing the mouth (wearing a mask), 2) clear pronunciation, 3) normal speech rate, 4) normal loudness, and 5) no noise (quiet private room). During the evaluation, participants were asked to adapt the speech speed, voice loudness, and noise according to their abilities when speech tracking. The difficulty level was adjusted on the basis of the speaker’s condition by showing and reading aloud the task text before starting, conducting phoneme training before starting, providing an overview of the task text, or providing hint words. After the training, the participants were given feedback regarding the words they had listened to again, heard properly, and made mistakes speaking. Auditory rehabilitation was conducted once a week (40 min per session) for 3 months. Assessments were conducted at the beginning and end of the training.

### Controls

We used data from the same subjects before and after the auditory rehabilitation intervention.

### Outcomes

Pure-tone audiometry and speech audiometry was performed with headphones using a conventional device (AA-78; Rion, Tokyo, Japan) in a soundproof room. Average hearing levels were calculated as the average of the measurements at 0.5, 1, 2, and 4 kHz obtained from pure-tone audiometry. Speech audiometry was conducted using a Japanese monosyllabic word list (67-S) that included 20 Japanese monosyllabic words developed by the Japan Audiological Society,^11^ and the speech discrimination score was defined as the maximum percentage of correct answers for stimuli equal to or under a sound level of 100 dB. The participants were evaluated using the speech-tracking rate (per minute using the speech-tracking method), Hearing Handicap Inventory for the Elderly (HHIE), Nursing Home Hearing Handicap Index (NHHHI), Token Test (new Japanese version), Communication ADL, Short Form (CADL), Kihon Checklist (KCL), Mini-Mental State Examination (MMSE), Digit Symbol Substitution Test (DSST), Symbol Search Test (from the Wechsler Adult Intelligence Scale-III), and word recall. These assessments are briefly described below.

The speech-tracking rate was calculated without a noise load or showing the mouth using the Mizonokuchi method with task text 1 for auditory rehabilitation assessment.

The HHIE is a questionnaire-based assessment in which participants self-rate the social and emotional impact of hearing loss by answering “yes,” “sometimes,” or “no” to 25 questions.^12,13^ The scores were 4 for “yes,” 2 for “sometimes,” and 0 for “no.” The total score was out of 100, and a higher score indicates a more severe hearing loss-induced handicap.

The NHHHI^14^ is a 10-item questionnaire with five choices, from 5 for “very often” to 1 for “almost never.” The total score is out of 50, and a higher score indicates a more severe hearing loss-induced handicap. The Token Test comprises parts A–E, in which the participants respond by pointing to a verb+object. Part F additionally includes elements such as nouns, verbs, adjectives, prepositions, conjunctions, and adverbs, and requires syntactic understanding. Responses are provided by manipulating tokens and are scored on the basis of the level of execution, with a higher score indicating better execution. A healthy distribution is assumed to be ≥160 points, and a typical cutoff is ≤158 points.^15^

The CADL^16^ assesses overall communication competence, including verbal and nonverbal functions. The scores range from 8.2 to 126.2, with higher scores indicating better function. The KCL is a self-administered questionnaire with 25 yes-or-no questions in seven domains: ADL, locomotion, undernutrition, oral functions, isolation, cognitive function, and depressed mood.^17^ The KCL is also used to assess frailty,^18^ which is a core symptom in older people, with a score of 8 or higher indicating frailty, 4–7 indicating pre-frailty, and 0–3 indicating a healthy state. The MMSE consists of 11 items: temporal awareness (time, place), memorization (repeating back item names), attention (calculation of serial 7’s or word reversal), recall (recalling item names), language (item names, sentence repetition, three-stage oral command, reading, writing), and copying a graphic.^19^ Scores ≥28 are in the normal range, scores of 24–27 indicate mild cognitive impairment, and scores of ≤23 indicate suspected dementia. In the DSST, the participant transcribes a symbol paired with a number, and the score is the number of symbols correctly transcribed in 120 s.^20^ The symbol search test consists of 60 questions. For each question, the subject scans for a sample stimulus symbol among a group of symbols, and answers “yes” or “no” as to whether the symbol was present. The number of correct and incorrect answers is recorded, with the raw score calculated as the number of incorrect answers subtracted from the number of correct answers on all pages. If the number of incorrect answers is greater than the number of correct answers, the raw score is zero.^20^ In the word recall test, participants are asked to list as many vegetables as they can, with five points allocated for listing ≥10 vegetables, 4 points for nine, 3 points for eight, 2 points for seven, 1 point for six, and 0 points for anything less than six. The test ends when the participants have given no answers for 10 s.

### Statistical analysis

Statistical analysis was performed using Statistical Analysis System version 9.3 (SAS Institute, Cary, NC, USA). Changes in scores of each assessment item and sub-item were examined using Wilcoxon’s signed-rank test, and the changes per question were examined using the sign test. The level of statistical significance was set at *p*<0.05.

## Results

After rehabilitation, 31 patients used HAs every day, whereas 28 patients used HAs every day before the rehabilitation.

**Figure 1:**
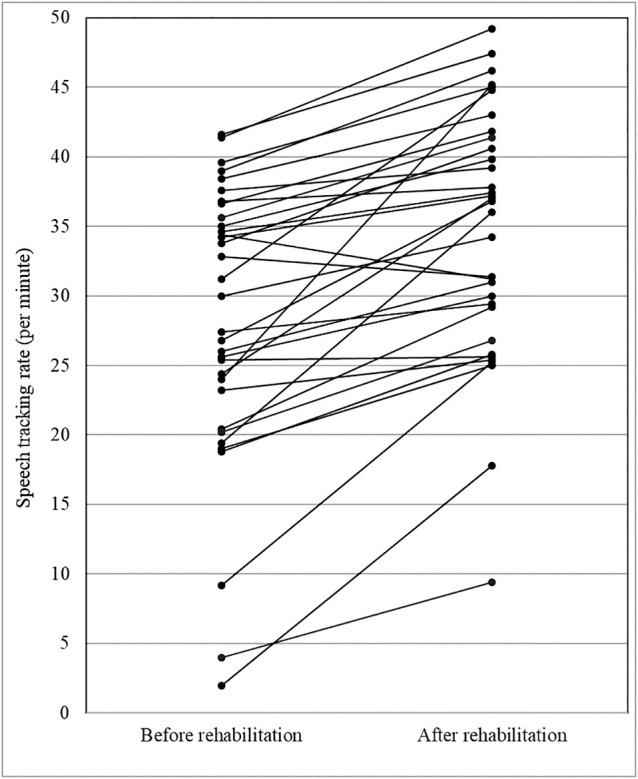
Change in tracking before and after auditory rehabilitation. The speech tracking rate per minute before and after intervention. Speech tracking rates improved in 31 of 33 participants after auditory rehabilitation. The speech tracking rate increased by up to 19.6 sentences per minute.

Figure 1 shows the changes in speech-tracking rate (*p*<0.001). The mean scores were 28.2 and 34.6 phrases/min at the start and end, respectively, with a mean improvement of 6.5 phrases/min. The speech-tracking rate improved in 31 participants (mean: 7.2 phrases/min), remained unchanged in one participant, and declined in one participant (mean: –2.2 phrases/min). The greatest increase was 19.6 phrases/min.

Table 1 shows the pre- and post-rehabilitation assessment item findings. The Token Test and MMSE results improved significantly. There was no difference between scores for parts A and E of the Token Test by site, with a significant increase in scores for part F after auditory rehabilitation in (*p*<0.001; Table 2). By item, the Token Test results were as follows: item 21 “point to both the small black square and the big red square” in part E (*p*=0.027), item 28 “please take a blue circle or a red square” in part F (*p*=0.007), item 31 “if there is a green circle here, please take a red square” in part F (*p*=0.009), and item 34 “all squares touch slowly, touch all circles quickly” (*p*=0.009), item 34 in part F (*p*=0.010), and item 36 “touch any circle except the black circle” (*p*=0.033) in part F. There was a significant difference in these items, and errors in particles and adverbs decreased. The distribution of cognitive function scores in the MMSE before auditory rehabilitation was as follows: 14 patients achieved a score ≥28, 12 patients achieved a score of 24–27, and seven patients achieved a score of ≤23. Hence, the MMSE score improved significantly (*p*=0.035). A significant number of participants showed improved scores for HHIE questions 5, 14, 15, and 21. The ratios of participants who improved to those who showed a decline in performance were 10 to 2, 11 to 3, 13 to 3, and 10 to 3 for questions 5, 14, 15, and 21, respectively. A significant number of participants showed improved scores for NHHHI questions 2, 4, and 10. The ratios of the participants who improved to those who showed a decline in performance were 10 to 2 for question 2, 10 to 2 for question 4, and 11 to 3 for question 10 (see Supplementary Material).

No significant change in KCL (*p*=0.413) scores was observed, but depression showed a tendency to improve from 1.6 to 1.2 (Table 3).

## Discussion

This study demonstrated a significant improvement in the speech-tracking rate, Token Test score, and MMSE score, but no change in the HHIE, NHHHI, CADL, KCL, or DSST scores; symbol finding; or word recall. Auditory rehabilitation using the Mizonokuchi method has three objectives, namely, to reconstruct appropriate auditory information with HAs and compensate for the loss of frequency and temporal resolution, to teach correct listening habits and communication strategies, and to train central cognitive information-processing skills.^8^

The speech-tracking method simultaneously assesses and trains listening and language comprehension, and involves interactions between the speaker and listener in ways that resemble actual communication situations. This method is effective for listening posture correction, such as facing the speaker’s direction and concentrating on listening, and provides immediate post-training feedback regarding which sounds were heard incorrectly. This method may be more effective for patients who find HAs ineffective and have difficulty wearing them. In the current study, the most common improvement among study participants was an increase in speech-tracking rate, which is consistent with previous reports of significant improvement in the no-noise condition after training.^21^ Because the speech-tracking method trains attention, comprehension, memory, speech, and auditory stimulation, it may have had a greater effect on cognitive function than auditory stimulation alone. However, less than 10% of audiologists provide hearing training to patients with hearing impairments.^22^ Along with supplementing hearing, SLHTs should consider providing auditory rehabilitation to improve and enhance listening and communication and help patients maximize the benefits of HAs, making their involvement in HA care worthwhile.

The speech tracking rate assessed in this study may vary depending on SLHT and other factors such as speech rate, inflection, dialect intonation, and eye contact. Moreover, the intimacy of the auditory rehabilitation process could vary, making the voice easier to hear. Therefore, there is a need to develop voice recordings and applications that minimize factors affecting speech-tracking rate assessments. Computer-based hearing-training methods have been reported in some countries, and in many cases, hearing skills improved using activities such as games.^23,24^ However, some studies have reported that auditory rehabilitation itself cannot be used reliably to assess the effects of the intervention on ADL, cognitive function, and language function in older people with hearing loss.^25^ Such interventions need to be studied further.

The increase in the Token Test score was thought to be caused by an improvement in auditory comprehension after training. Although the improvement at the end of the training may have been caused by familiarity with the test situation, the increase in the frequency of communicative situations caused by the weekly 1-hour conversation and training during auditory rehabilitation, and the fact that training required the participants to track their speech correctly may also have contributed to the improvement. In addition, the training required correct speech tracking, which led to fewer errors with particles and adverbs in each section (parts E and F), leading to higher scores. Therefore, it is possible that awareness of listening to the details of the text was effective in improving performance on the auditory comprehension task.

Although the total MMSE score significantly improved, the subscale and word recall scores did not significantly change, possibly because of low power as a result of the small sample size. Older adults with no hearing loss compensation have been reported to be unable to recall as much detail as younger adults in both quiet and noisy environments, indicating that speech comprehension difficulties in older adults primarily reflect a decline in hearing rather than a decline in cognitive ability.^26^ Although proper HA fitting can lead to consistent use and training to listen correctly is effective, more research is needed in this area. As reported in a previous study,^3^ although our patients’ HHIE scores indicated a need for HAs, they had low awareness of their impairment and tended to underestimate their hearing loss. After 120 days of training at reading aloud and writing Kanji characters and 15 min of reading newspaper articles, positive changes, such as improvements in pure tone and speech audiometry after 10 months and the ability to actively interact with others in daily life, were reported.^27^ In a study of 94 hearing-impaired older adults without cognitive decline, HHIE scores improved from 30.8 to 18.0 after HA use, with significant improvements in scores for 18 questions.^28^ However, more than half of the patients in this study (19 of 33) were cognitively impaired, suggesting that their understanding of hearing loss may not have changed significantly from the beginning to the end of the study. Nevertheless, hearing the television and radio (HHIE question 15 and NHHHI question 4) showed significant subjective and objective improvements. Emotional aspects involving family members (HHIE questions 5 and 14 and NHHHI question 2) also showed significant improvements.

The CADL scores did not significantly differ, with mean scores of 115.8 and 118.6 at the start and end of the study, respectively, both of which were close to the perfect score, suggesting a ceiling effect because participants had already reached communication level 5. The results of this study are consistent with those of our previous study, which demonstrated no significant change in total KCL scores before and after auditory rehabilitation and no significant change in total KCL scores before and after the introduction of HAs.^29^ In the KCL sub-items, depression showed a tendency to improve, and the rehabilitation may have enhanced self-esteem. Processing speed when looking for signs and symbols was assessed to examine for changes as auditory processing improved as a result of the rehabilitation. Although a significant DSST score improvement after HA use has been reported,^28^ we observed no significant changes. Most participants exhibited cognitive decline and impaired attention sustenance, selectivity, and distribution. Thus, auditory rehabilitation may have been inadequate for improving processing speed.

A novel finding in this study is that auditory rehabilitation using the speech-tracking method in combination with HA use improved auditory comprehension in older people with hearing loss. However, some of the changes after auditory rehabilitation may have been caused by learning effects. It is possible that there was a selection bias in that the group was highly motivated to wear HAs and participate in the rehabilitation, which may have affected the results. This study was a preliminary study with inclusion criteria to select older patients whose speech discrimination was less than 70% and who had difficulty using an HA. There were no exclusion criteria. Therefore, this study included those who had already worn HAs and those who had never worn HAs, and those who wore HAs in the better ear, worse ear, and both ears. In addition, not all participants were able to wear their HAs every day. Because the duration of HA use was not studied, it was not possible to investigate changes in HA use in detail. A more systematic study with a control group is needed.

## Figures and Tables

**Table1:** Pre-and post-rehabilitation assessment item scores

	Before hearing rehabilitation	After hearing rehabilitation	*p* value*
Speech tracking rate (per minute)	28.2±10.1	34.6±9.1	<0.001
HHIE	37.9±24.6	33.3±25.0	0.057
NHHHI	29.4±14.6	27.3±13.4	0.116
Token Test	148.8±12.4	157.6±8.2	<0.001
CADL	115.8±13.0	118.6±10.9	0.239
KCL	6.3±3.8	5.7±3.6	0.413
MMSE	26.1±3.3	26.9±3.0	0.035
Digit symbol substitution test	46.5±16.0	47.9±17.3	0.518
Symbol search	23.7±8.0	24.4±9.1	0.370
Word recalling	4.2±1.5	4.2±1.6	0.710

^a^Wilcoxon signed-rank test HHIE, Hearing Handicap Inventory for Elderly; NHHHI, Nursing Home Hearing Handicap index; CADL, Communicative Abilities in Daily Living; KCL, Kihon Checklist; MMSE, Mini-Mental State Examination

**Table2:** Pre-and post-rehabilitation Token Test score

	Before hearing rehabilitation	After hearing rehabilitation	*p* value*
A	7.0±0.0	7.0±0.2	1.000
B	7.9±0.2	7.9±0.3	1.000
C	11.7±0.8	11.8±0.7	0.641
D	15.7±0.8	15.8±0.4	0.227
E	23.0±1.9	23.2±1.4	0.996
F	83.5±10.5	91.9±7.0	<0.001

^a^Wilcoxon signed-rank test

**Table3:** Pre-and post-rehabilitation KCL item scores

	Before hearing rehabilitation	After hearing rehabilitation	*p* value*
ADL	1.3±1.5	1.4±1.5	0.575
Life function	1.2±1.0	1.4±1.1	0.446
Nutritional status	0.0±0.0	0.1±0.3	untestable
Oral function	0.9±0.7	0.8±0.8	0.807
Social ADL	0.3±0.5	0.4±0.5	0.500
Cognitive function	0.7±0.8	0.5±0.7	0.145
Depressive mood	1.6±1.3	1.2±1.3	0.084

*Wilcoxon signed-rank test ADL, activities of daily living

## Data Availability Statement

The data that support the findings of this study are available on request from the corresponding author.

## Clinical Trial Registration

This study was not registered with the University Hospital Medical Information Network Clinical Trials Registry.
